# Comparison of the effects of virtual training by serious game and lecture on operating room novices’ knowledge and performance about surgical instruments setup: a multi-center, two-arm study

**DOI:** 10.1186/s12909-022-03351-5

**Published:** 2022-04-11

**Authors:** Fakhridokht Akbari, Morteza Nasiri, Neda Rashidi, Sahar Zonoori, Leila Amirmohseni, Jamshid Eslami, Camellia Torabizadeh, Fahimeh Sadat Havaeji, Marzieh Beigom Bigdeli Shamloo, Crislaine Pires Padilha Paim, Mehran Naghibeiranvand, Masoomeh Asadi

**Affiliations:** 1 Department of Nursing, Behbahan Faculty of Medical Sciences, Behbahan, Iran; 2 Student Research Committee, Shiraz University of Medical Sciences, Shiraz, Iran; 3 Department of Operating Room Nursing, School of Nursing and Midwifery, Shiraz University of Medical Sciences, Shiraz, Iran; 4 Department of Operating Room Technology, School of Paramedical Sciences, Dezful University of Medical Science, Dezful, Iran; 5 Department of Nursing, Broujerd School of Nursing, Lorestan University of Medical Sciences, Khormaabad, Iran; 6 Department of Operating Room Nursing, Behbahan Faculty of Medical Sciences, Behbahan, Iran; 7 Department of Nursing, School of Nursing and Midwifery, Shiraz University of Medical Sciences, Shiraz, Iran; 8 Department of Operating Room Technology, School of Paramedical Sciences, Qom University of Medical Sciences, Qom, Iran; 9 Department of Clinical Nursing, Nursing and Midwifery School, Ahvaz Jundishapur University of Medical Sciences, Ahvaz, Iran; 10 Department of Graduate Nursing Program, Institute of Cardiology of Rio Grande Do Sul, University Foundation of Cardiology, Porto Alegre, Brazil; 11 Department of Nursing, Islamic Azad University, Khorramabad Branch, Khorramabad, Iran; 12 Department of Operating Room Nursing, Abadan University of Medical Sciences, P.O. Box 6313833177, Abadan, Iran

**Keywords:** Lecture, Perioperative nursing, Serious game, Surgical instruments

## Abstract

**Background:**

Game-based training is increasingly implemented in different nursing fields, as it allows students to learn experientially, with the flexibility to regulate their training based on their personal progresses and abilities. This study aimed to compare the effects of virtual training by the *“Playing with Surgical Instruments (PlaSurIn)”* game and the lecture on the surgical instruments setup knowledge and performance of Operating Room (OR) novices.

**Methods:**

This study was conducted on 51 s-semester undergraduate OR technology students taking the course “An Introduction to Surgical Instruments and Equipment.” An additional virtual training session was held via a learning management system using two different methods. The students of the Game Training Group (GTG, *n* = 27) played individually with the *“PlaSurIn”* game during a week, while the students of the Lecture Training Group (LTG, *n* = 24) received the lecture-based training during a week. To measure knowledge, all the students participated in a theoretical test with 10 multiple-choice questions before and immediately after the training. They also participated in an Objective Structured Clinical Examination (OSCE) after the training, and their performance was evaluated by the remained time for setup completion and the scores, errors, and bonuses.

**Results:**

The mean score of the theoretical test was significantly higher in the GTG than in the LTG after the training (*p* = 0.040). Additionally, the GTG participants had higher scores (*p* = 0.016), fewer errors (*p* = 0.001), and higher bonuses (*p* = 0.011) compared to the LTG ones. The remained time for setup completion was also significantly longer in the GTG than in the LTG (*p* < 0.001).

**Conclusion:**

Virtual training by *“PlaSurIn”* was superior to the lecture-based method for the enhancement of surgical instruments setup knowledge and performance amongst OR novices.

## Background

A complex issue in perioperative nursing training is the way novices develop their technical or procedural skills, improve their performance, and acquire their theoretical or practical knowledge [1]. Most perioperative nursing training occurs through the clinical apprenticeship methods in surgical wards and Operating Rooms (ORs) [2]. In these learning environments, trainees experience high levels of stress due to being subjected to time pressures by the OR personnel, which makes them susceptible to clinical errors [3]. In addition, OR trainees face challenges regarding traditional training due to the inherent nature of perioperative nursing, increasing demands to improve patients’ safety, limitations in OR resources, and restrictions in perioperative trainers’ work hours [1–3]. Accordingly, it is necessary to complement or change the traditional training methods to access the required perioperative nursing competencies among OR novices.

Game-based training, as an alternative method for traditional training, has received increasing attention in different nursing fields [4, 5]. Serious games developed in perioperative nursing allow trainees to acquire knowledge and develop skills in a safe and relaxed virtual learning environment [6, 7]. These games create a fun and positive interactive learning experience for trainees and foster their collaboration, communication, and critical thinking by assisting them to maintain their engagement in training, decrease their fear of unknown and environmental stress, and prevent their clinical errors, ultimately improving the quality and safety of patient care [4, 8].

**Fig. 1:**
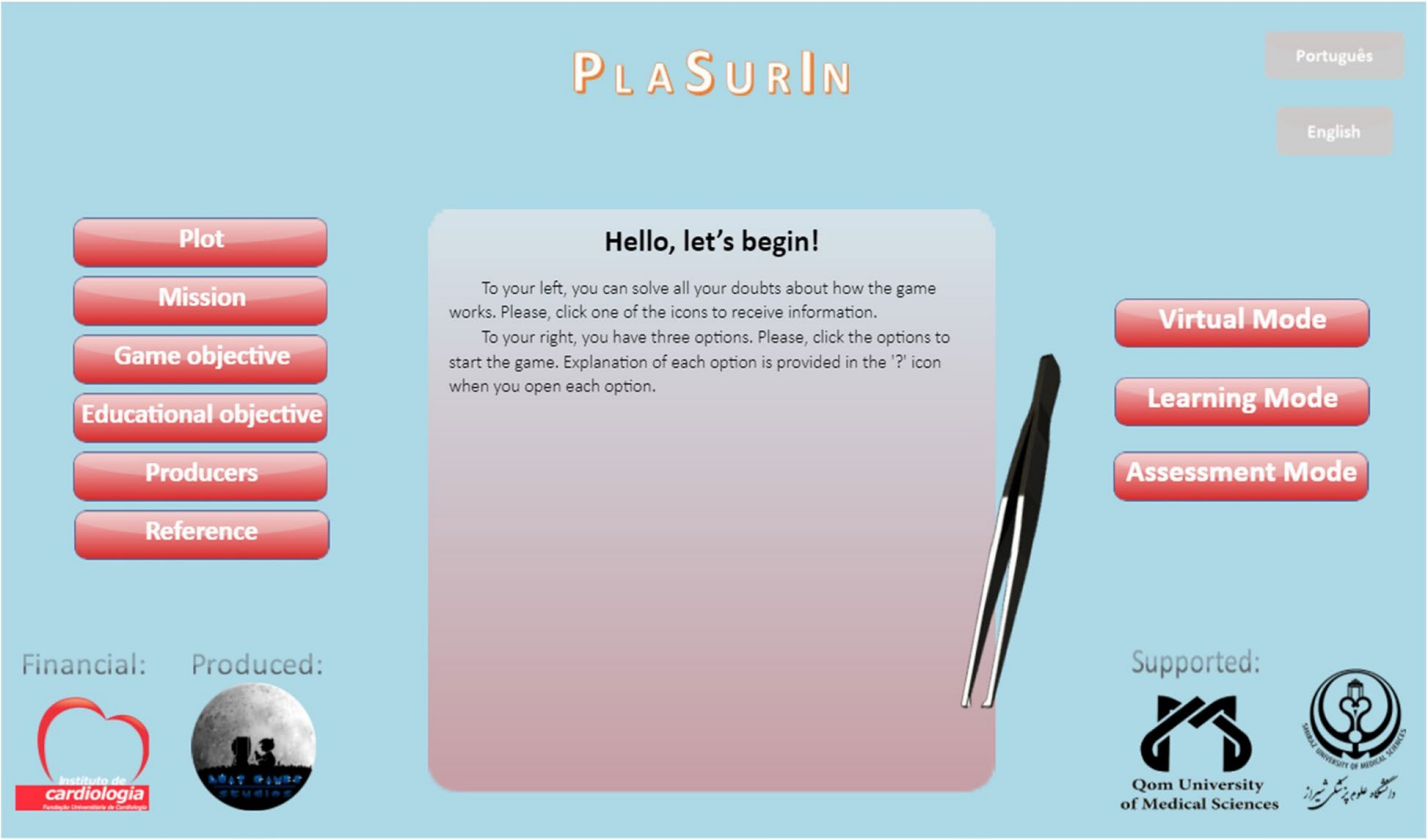
A screenshot of the main menu of the *“Playing with Surgical Instruments (PlaSurIn)”* game

**Fig. 2:**
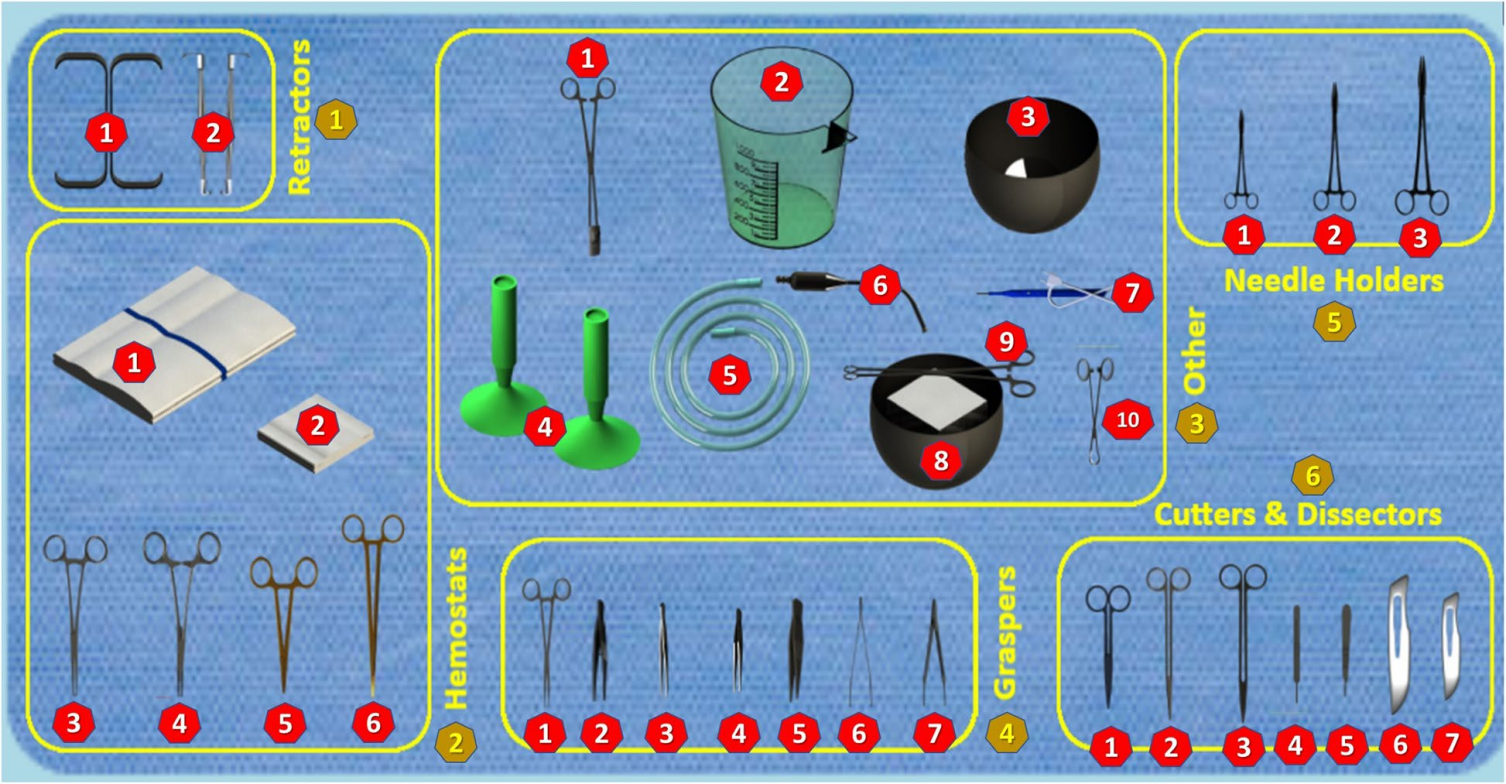
A screenshot of the “virtual mode” of the *“Playing with Surgical Instruments (PlaSurIn)”* game: each classification as well as the instruments have been numbered for recording the obtained scores and number of errors in the objective structured clinical examination

**Fig. 3:**
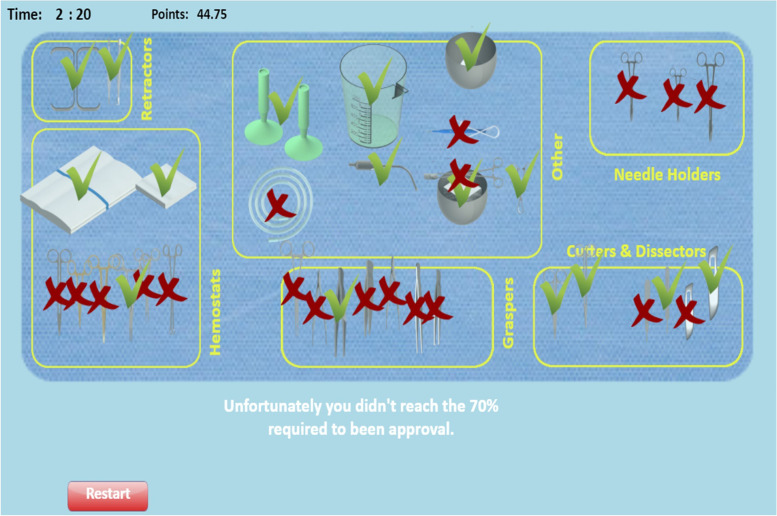
A screenshot of the “assessment mode” of the *“Playing with Surgical Instruments (PlaSurIn)”* game after playing the game

Among different games developed in perioperative fields, considerable attention has been devoted to games in the field of surgical instrumentation. In this regard, different types of games have been introduced in nursing and medical training such as *“Playing with Tweezers”* [9], *“Play and Learn for Surgeons”* [10], *“PeriopSim™ instrument trainer”* [11], and *“Nintendo Wii U™”* [12–14]. Recently, a serious game called *“Playing with Surgical Instruments (PlaSurIn)”* was developed for OR novices to set up basic surgical instruments on the Mayo stand or a back table at the commencement of minor general surgeries [15]. The *“PlaSurIn”* is an English computer-based game adapted from a Portuguese game called the *“Playing with Tweezers”* through a rigorous modification approach and validation process [9, 15]. The *“PlaSurIn”* has a “main menu” containing three main options on the right side, namely “virtual mode,” “learning mode,” and “assessment mode” (Fig. 1). In the “virtual mode,” trainees can view the setup of 35 instruments in the designed table, six instrument classifications (i.e., retractors, hemostats, others, graspers, cutters and dissectors, and needle holders), and each instrument’s information and image when clicking on the instrument (Fig. 2). In the “learning mode,” trainees set up instruments on the designed table according to the “virtual mode” as many times as needed and can request tips if necessary. In the “assessment mode,” trainees recreate the instruments setup according to the platform of the “learning mode” in seven minutes. They can also see their obtained scores and the remained time for setup completion at the end of the “assessment mode.” Moreover, they receive a bonus at the end of the “assessment mode” in case they place all the instruments of a classification in their correct positions on the designed table. Trainees must reach the appropriate level of 70% regarding the correct positions of the instruments in each attempt; otherwise, they should repeat the game whenever they wish to promote their performance (Fig. 3) [15].

In a recent systematic review, games used in health professions were reported to be effective as a training method. Many studies also indicated that games were more effective in improving the trainees’ knowledge and performance [16]. Although the *“PlaSurIn”* game has been validated, the effects of playing with this game compared to other training methods on learning outcomes have not been evaluated [15]. Considering the importance of comparative studies for drawing evidence-based conclusions regarding the superiority of serious games over other types of training strategies, the present study aims to compare the effects of virtual training by the “*PlaSurIn”* game and the lecture on surgical instruments setup knowledge and performance amongst second-semester undergraduate OR technology students. We assumed that virtual training by the *“PlaSurIn”* game is more effective than virtual training by the lecture-based method in enhancing surgical instruments setup knowledge and performance.

## Methods

### Setting and participants

The present study was conducted at Abadan University of Medical Sciences (AUMS) and Shiraz University of Medical Sciences (SUMS). Undergraduate OR technology students were invited during the academic year 2020–2021 to participate in the study. The inclusion criteria were 1) taking the course “An Introduction to Surgical Instruments and Equipment,” 2) studying in the second semester, and 3) having access to a personal computer. The students were excluded if they had any experience of participating in the surgical instruments setup courses or playing serious games. Totally, 51 students were invited. All these students met the inclusion criteria and willingly accepted to participate in the study. To avoid sharing information between the students of two groups, the conditions were randomly assigned to universities by coin tossing. To this end, 24 students who studied at AUMS were allocated to the Lecture Training Group (LTG), and 27 students who studied at SUMS were assigned to the Game Training Group (GTG).

### Intervention

According to the program of the Ministry of Health and Medical Education in Iran, the course “An Introduction to Surgical Instruments and Equipment” contains two theoretical credits, which is equal to 16 two-hour sessions. Since surgical instruments setup is trained by clinical apprenticeship methods, less attention is paid to these topics in this course. Accordingly, an additional session was conducted to train the students how to set up basic surgical instruments on the Mayo stand or a back table in minor general surgeries.

The course was presented in 16 theoretical sessions in the second semester by the corresponding professor, and the same course plan, lesson plan, training approach, and evaluation method were implemented for the students of the two groups. Moreover, the curriculum was the same between groups during the study and also before the study. In two groups, the same topics regarding the names and applications of surgical equipment and instruments were trained via a Learning Management System (LMS). This virtual training method has been routinely performed at most universities in Iran during the Coronavirus Disease 2019 (COVID-19) pandemic. For each session, a two-hour lecture was presented and the students were provided with educational resources (i.e., multimedia, textbooks, and podcasts), assignments, and self-exams with Multiple-Choice Questions (MCQs). At the end of the semester, the final theoretical test with MCQs was conducted through an online synchronous system for the students of two groups under the same conditions.

Four weeks after the final theoretical test, an additional session was conducted to train students how to set up basic surgical instruments. This session was held for both groups during a week by an experienced professor who was unaware of the study objectives using either game-based or lecture-based training. The students of both groups were informed that top-performing students in the additional session would receive prizes to ensure their maximum performance.

The lecture-based training was performed by using the LMS in the same approach as the theoretical sessions. To this end, a two-hour online session based on lecture and Microsoft PowerPoint presentation was held on the first day of the additional session, and the students were trained the principles of surgical instruments setup in minor general surgeries similar to the platform established in the “virtual mode” of the *“PlaSurIn”* game. The students were also given the last edition of the book “Surgical Mayo Setups” [17] and a recorded video containing the instructions presented in the online session and they were allowed to review them as much as they wished during a week. Moreover, some assignments and self-exams were presented and the students were requested to do them individually during the week. In addition, the students were allowed to send messages to their professor when needed through the “messages module.” Afterwards, all the functions provided via the LMS, except for the “messages module,” were deactivated. At the end of the week, no message was received from the students.

Game-based training was defined as playing with the *“PlaSurIn”* game. First, a video clip was presented in the LMS to instruct the students how to play the game. Then, the game Uniform Resource Locator (URL) address was presented and the students were provided with unique usernames and passwords to enter the game. They were requested to play the game individually on the computer whenever they wished to do so during a week. Afterwards, the game URL address and the related video clip were deactivated. The students were also asked to send messages to their professor through the “messages module” in order to resolve their problems about the game. In total, three students reported their problems during the week, which were mainly due to invalid usernames or passwords. Other functions of LMS (i.e., online session, assignments, and self-exams) were not used for the students in the GTG.

### Study outcomes

To evaluate the homogeneity of the groups in terms of the demographic and educational data, the following information was recorded: age, gender, marital status, previous semester’s Grade Point Average (GPA, average grade received for all the courses a student took in the previous semester), number of credits taken in the current semester, and the final theoretical test score of the course “An Introduction to Surgical Instruments and Equipment.”

To assess the students’ knowledge of the basic surgical instruments setup on the Mayo stand or a back table at the commencement of minor general surgeries, a theoretical test was performed. This test consisted of 10 MCQs (e.g., item No. 1: on which part of the table do you place a cutter or dissector instrument) developed by five researchers (instructors of surgical instruments). The qualitative content validity of the test was approved by 10 OR faculty members and its qualitative face validity was satisfactory as determined by 10 students with similar characteristics to those of the target population. In order to evaluate the internal consistency and stability of the test, a test–retest analysis was done with a 12-day interval. In doing so, 30 OR technology students who were not supposed to participate in the main analysis took part in the test, and satisfactory results were obtained by Kuder-Richardson 20 (KR20, *r* = 0.76) and Intra-Class Correlation (ICC, *r* = 0.81) [18]. The theoretical test was performed for the students of two groups in 15 min on the first and last days of the additional session through an online synchronous system under the same conditions. To decrease the possibility for random answering, correct answers were displayed at the end of the test and the order of questions was set randomly. The students’ scores were recorded by a blinded professor. It is worth mentioning that scores one and zero were assigned to correct and wrong answers, respectively. Therefore, the total score range was from 0 to 10, with higher scores indicating greater knowledge of the surgical instruments setup.

The students’ performance on the surgical instruments setup on the Mayo stand was evaluated by an Objective Structured Clinical Examination (OSCE) one week after the end of the additional session. The OSCE was performed in a single station in the corresponding university under the same conditions. Accordingly, the students were asked to set up instruments on the Mayo stand within seven minutes. This time was considered based on the time allocated in the “assessment mode” of the *“PlaSurIn”* game. Also, to ensure that the time allocated for OSCE was adequate, a pilot OSCE was run before the real OSCE for four second-semester OR technology students, who were not included in the main analysis. During the OSCE, a blinded rater recorded the students’ skills using an OSCE form, which addressed the four items of time, score, error, and bonus. In this regard, time, score, and bonus were considered based on the variables presented in the *“PlaSurIn”* game [15], while error was regarded as a new variable in the current study. Time was considered as the remained time for setup completion. Score was defined as the total score obtained out of 100 by summing up the instrument’s classification scores calculated by multiplying the number of instruments in a classification by the importance score of that classification, as follows: 1) retractors: 2 × 2.5 = 5, 2) hemostats: 6 × 2.75 = 16.5, 3) others: 10 × 2.75 = 27.5, 4) graspers: 7 × 3 = 21, 5) cutters and dissectors: 7 × 3 = 21, and 6) needle holders: 3 × 3 = 9. Error was considered the total number of errors in setting up the instruments in their correct positions, which could range from 0 to 35 **(**Fig. 2). Finally, bonus was defined as the correct positioning of all the instruments in a classification. Considering the six classifications, the number of bonuses could range from zero to six.

The qualitative face validity and content validity of the OSCE form were approved by 10 OR faculty members. In addition, its inter-rater reliability was measured by calculating the correlation between the scores recorded by two trained OR faculty members with the same professional characteristics for 30 OR technology students (not participating in the main analysis). The ICC coefficients for the agreement between the faculty members were found to be 0.81–0.92, which signified the substantial agreement of the ratings [19]. To avoid bias, the rater who completed the OSCE form at each university was trained by the main researcher how to evaluate the students’ performance. The ICC coefficients for the agreement between the raters were 0.86–0.91.

### Ethical consideration

The study was approved by the Regional Research Ethics Committee of AUMS. All methods were performed in accordance with the relevant guidelines and regulations. The study was also reported based on Guideline for Reporting Evidence-based practice Educational interventions and Teaching (GREET) [20].

### Statistical analysis

The data were analyzed using the SPSS software, version 22 (SPSS, IBM^©^ Corp., Armonk, NY, USA), and *p* < 0.05 was considered statistically significant. The normal distribution of the quantitative data was not confirmed by the Kolmogorov–Smirnov test. Therefore, the homogeneity of the study groups in terms of demographic and educational characteristics was assessed using Mann–Whitney U test and Fisher’s exact test. Additionally, Mann–Whitney U and Wilcoxon signed-rank tests were used for within-group and between-group comparisons of the students’ theoretical test scores. Mann–Whitney U test was also employed to compare the study groups concerning the OSCE score.

## Results

### Demographic characteristics

All the students in the two groups were single. The results also revealed no significant difference between the two groups in terms of other demographic and educational data (Table 1).

**Table 1:** Demographic and educational characteristics of the operating room technology students in the study groups

Variables	Intervention group^a^ (*n* = 27)	Control group^b^ (*n* = 24)	Test results	*P*
Age (years)	19.59 ± 0.57	19.79 ± 0.50	261.00^**c**^	0.126
Gender				
Female	11 (40.7)	16 (66.7)	Fisher's exact test	0.093
Male	16 (59.3)	8 (33.3)		
Previous semester’s grade point average (range: 0–20)	17.12 ± 1.25	17.06 ± 1.51	301.50^**c**^	0.671
Number of credits taken in the current semester	19.18 ± 4.26	18.45 ± 1.91	323.00^**c**^	0.985
Final theoretical test score of the course “An Introduction to Surgical Instruments and Equipment” (range: 0–20)	15.01 ± 1.30	15.99 ± 1.83	240.00^**c**^	0.112

All values have been expressed as mean ± standard deviation or number (percentage)

^a^ Played with the *“Playing with Surgical Instruments (PlaSurIn)”* game individually during a week

^b^ Received virtual lecture-based training during a week

^**c**^ Mann–Whitney U test

### Knowledge

The results showed no significant difference between the study groups regarding the mean score of the theoretical test at baseline (*p* = 0.306). However, the mean score obtained by the students in the GTG was significantly higher than that obtained by the students in the LTG after the training (*p* = 0.040) (Table 2). The two groups’ mean scores were significantly higher after the training compared to the baseline (*p* < 0.001 in GTG, *p* = 0.010 in LTG).

**Table 2:** Surgical instruments setup knowledge and performance of the operating room technology students in the study groups

Variables			Intervention group^a^ (*n* = 27)	Control group^b^ (*n* = 24)	Test results^c^	*P*
Knowledge (range: 0–10)	Before the intervention		4.00 ± 1.79	4.58 ± 2.10	270.50	0.306
	After the intervention		6.70 ± 0.72	5.91 ± 1.71	223.50	0.040
Performance	Remained time for setup completion (min)		2.00 ± 0.65	1.11 ± 0.95	131.50	< 0.001
	Score (range: 0–100)		91.95 ± 1.33	89.94 ± 2.78	198.00	0.016
	Error number	2	7 (25.9)	3 (12.5)	150.50	0.001
		3	20 (74.1)	8 (33.3)		
		4	0 (0.0)	9 (37.5)		
		5	0 (0.0)	3 (12.5)		
		6	0 (0.0)	1 (4.2)		
	Bonus number	1	0 (0.0)	1 (4.2)	196.00	0.011
		2	0 (0.0)	5 (20.8)		
		3	11 (40.7)	9 (37.5)		
		4	7 (26.0)	7 (29.2)		
		5	9 (33.3)	2 (8.3)		

All values have been expressed as mean ± standard deviation or number (percentage)

^a^ Played with the *“Playing with Surgical Instruments (PlaSurIn)”* game individually during a week

^b^ Received virtual lecture-based training during a week

^**c**^ Mann–Whitney U test

### Performance

The remained time for setup completion was significantly longer in the GTG than in the LTG (*p* < 0.001). The scores and number of bonuses were also significantly higher in the GTG compared to the LTG (*p* = 0.016, *p* = 0.011). On the other hand, a significantly fewer number of errors were made by the students in the GTG compared to those in the LTG (*p* = 0.001) (Table 2).

## Discussion

This study aimed to compare the effects of virtual training by the *“PlaSurIn”* game and lecture-based method on the surgical instruments setup knowledge and performance of OR novices. The results demonstrated that the remained time for the setup completion was significantly longer in the students trained by the *“PlaSurIn”* game in comparison to those trained by the lecture. In addition, the students in the GTG had fewer errors and obtained higher scores and bonuses compared to those in the LTG. The results also indicated that the students in the GTG gained higher theoretical test scores compared to those in the LTG after the training. Although both groups’ theoretical test scores were higher after the intervention compared to the baseline, the increase was more prominent in the GTG. Hence, the results supported the hypothesis that using the *“PlaSurIn”* could enhance the surgical instruments setup knowledge and performance among OR novices.

The *“PlaSurIn”* has been introduced as the first international game of surgical instruments setup developed in the nursing field. Previous studies regarding the effects of game-based training in surgical instrumentation are limited to medical students and surgical residents [11–14, 21]. The results of a recent study showed the effectiveness of *“Play and Learn for Surgeons*,*”* a serious game in uterine artery ligation surgery, on the surgical instruments handling and knowledge of surgical instruments amongst obstetrics and gynecology residents [10]. The results of another study indicated that using the *“PeriopSim™ instrument trainer”* and *“PeriopSim™ for burr hole surgery”* significantly increased the total score, saved time, and decreased errors in the identification of neurosurgical instruments during a simulated burr hole surgery procedure among neurosurgery residents [11]. Similarly, playing with *“Nintendo Wii U™,”* a laparoscopic video game, could improve inexperienced medical students’ performance regarding laparoscopic instrument handling [12–14]. Although the findings of the reviewed studies are in line with those of the current study concerning the beneficial effects of serious games on surgical instrumentation skills, the comparison should be done with caution due to the differences in the populations studied, game objectives and designs, and methods used to evaluate the trainees’ performance.

### Study implications

Based on the findings, it seems that the *“PlaSurIn”* game could be used as an acceptable alternative or complement to the traditional training methods to access the required knowledge and performance about surgical instruments setup among second-semester OR technology students. Given that the *“PlaSurIn”* game is free of cost and has an easy-to-use platform, it can be used in future training programs to enhance OR novices’ surgical instruments setup knowledge and performance in a limited amount of time with reduced educational resources, especially when there is no access to routine training methods including the COVID-19 lockdown period. Moreover, the *“PlaSurIn”* game is of practical use when perioperative trainers are under pressure due to restrictions in their work hours and a large number of students.

The results of this study could be generalized to other universities with similar student demographics. Moreover, the *“PlaSurIn”* game could be used to train surgical instruments setup for other health-related students such as inexperienced nurses and surgical residents during their OR apprenticeship courses. However, further studies are required to evaluate the generalizability of the present findings to other health-related students. Accordingly, it is hoped that similar interventions will be implemented and tested amongst other novices, who are involved in setting up surgical instruments, to evaluate the generalizability of the present findings to other educational settings and participants.

### Study strengths

To the best of our knowledge, this work was the first interventional study to evaluate an educational game on the surgical instruments setup in the nursing field. One major strength of this study was the evaluation of both knowledge and performance. In doing so, OSCE was used since it has been reported to be a well-validated evaluation approach [22]. This was, in fact, another strength of the current study since most previous studies evaluated trainees’ performances using simulations such as the virtual reality. Additionally, most previous studies on this topic did not assess the validity and reliability of their measures. In the current study, however, a group of OR faculty members and students confirmed the face and content validity of the theoretical test and the OSCE form. The reliability of these tools was confirmed, as well. Moreover, the OSCE raters were instructed regarding the assessment tool and their agreement was satisfactory.

### Study limitations

The study findings should be interpreted with caution due to some limitations. Even though the students were recruited from two universities via census, the small sample size in each group can limit the generalizability of our findings. In addition, generalization of the present findings can be limited as group allocation was done based on the university, which might lead to selection bias due to the inadequate generation of a randomized sequence. Moreover, no pretest was performed for performance, as it could lead to a bias related to the use of the same OSCE. Likewise, this study did not measure whether the students were able to retain the knowledge learned from the two training methods. Finlay, the findings could be affected by inequality in the students’ computer literacy. However, the students in two groups were instructed how to use the LMS so as to minimize this difference.

## Conclusions

Training by the *“PlaSurIn”* game was more effective compared to the lecture-based method in the improvement of the surgical instruments setup knowledge and performance of OR novices. Thus, it seems that the *“PlaSurIn”* game can contribute to the development of OR novices’ skills for surgical instruments setup. However, further long-term studies with larger sample sizes are recommended in this area.

## Data Availability

Data and materials are available by contacting the corresponding author.

## Abbreviations

AUMS: Abadan university of medical sciences
COVID-19: Coronavirus disease 2019
GPA: Grade point average
GREET: Guideline for Reporting Evidence-based practice Educational interventions and Teaching
GTG: Game training group
ICC: Intra-class correlation
KR20: Kuder-Richardson 20
LMS: Learning management system
MCQ: Multiple-choice questions
OR: Operating room
OSCE: Objective structured clinical examination
LTG: Lecture training group
PlaSurIn: *“Playing with Surgical Instruments”* Game
SPSS: Statistical package for social sciences
SUMS: Shiraz university of medical sciences
URL: Uniform resource locator
